# Supplementary material for: Are carbon emissions trading and green financial instruments synergistic? -Comprehensive quantitative research based on content analysis
Source: PLoS One. 2024 Mar 7;19(3):e0298601. doi: 10.1371/journal.pone.0298601 (PMC10919601; doi:10.1371/journal.pone.0298601)
Supplement: S1 Data — (ZIP) [file pone.0298601.s001.zip › Data/地级市Z_F工作报告.zip!/╕╩╦α/└╝╓▌╩╨/└┤╫╘╣1⁄2╓┌║┼í╛┬φ┐╦╩2╛▌═°í┐_2014.docx]

各位代表：

现在，我代表市人民政府向大会作工作报告，请予审议，并请市政协委员和其他列席人员提出意见。

2013 年工作回顾

过去的一年，在省委、省政府和市委的正确领导下，我们认真贯彻落实党的十八大以来各项重大部署和习近平总书记系列重要讲话精神，按照省上“做快新区、做大产业、做美城市、做实民生” 的要求，着力实施“3341”项目工程，统筹推进各项重点工作，加快建设全国有影响力的区域性特大城市，全市经济社会发展呈现增速较快、难点突破、民生改善、和谐稳定的良好局面。

（一）经济首位度和综合实力逐步提高。全市实现生产总值 1776.83 亿元，增长 13.4%，在全省的首位度为 28.2%，比上年提高 0.51 个百分点。固定资产投资 1623.71 亿元，增长31.03%。社会消费品零售总额 843.87 亿元，增长 14.7%公共财政预算收入 124.52 亿元，增长 20.04%；公共财政预算支出242.32 亿元，增长 19.61%。进出口总额 40.6 亿美元，增长19.63%。节能减排任务全面完成，万元生产总值能耗下降4.46%。“3341”项目工程入库项目 1984 个，计划总投资 8734亿元，开工建设亿元以上项目 872 个。市列 100 个重大项目完成投资 532.49 亿元。全市金融机构存贷比达到 85.42%，创历史新高，新增存款和贷款分别占到全省的 46.81%和 44.66%。兰州银行存贷款业务大幅增长，成为区域内同业存贷款规模 最大的商业银行。市财政安排专项引导资金 3000 万元，支持中川机场业务扩展，机场旅客吞吐量达到 560 万人次，增幅位居全国省会城市第一，成为国内大型运输机场和国际航空口岸。兰渝铁路、兰新铁路第二双线、宝兰客运专线、西客站等重大工程加快建设，兰州的交通枢纽地位正在加速提升。认真研究国家差别化区域经济政策，向上争取各类资金93.89 亿元，获批了兰白经济区承接产业转移示范区、信息消费试点城市、智慧城市试点、新能源汽车推广应用城市、公交都市示范城市、中国百合之都、兰州新区科技创新城、东部科技新城新型城镇化示范区等国家级和省级政策平台。

（二）产业结构在调整中趋于优化。全市实现一产增加值 49.67亿元，增长6.7%； 二产增加值820.42 亿元，增长 13.5%；三产增加值 906.74 亿元，增长 13.6%。三次产业比例调整为 2.8∶46.17∶51.03，整体上呈现布局调整、结构优化、转型升级的良好趋势。积极贯彻省上“365”现代农业发展行动计划，制定了农村富民多元产业发展规划，出台设施农业、肉羊产业、土地流转“三个 1000 万”扶持办法，新增设施农业 1.35 万亩、肉羊16.2 万只、土地流转 13.9 万亩，中药材、玫瑰、百合等特色农产品种植面积达到 116.84 万亩，高原夏菜产量达到 251 万吨。坚持传统产业改造升级与新兴产业培育两手抓，完成工业固定资产投资 469.21 亿元，增长 35.28%，实现工业增加值614.45 亿元，增长 14.1%，扭转了工业固定资产投资增速低于全社会固定资产投资增速的局面，遏制了工业增加值占全省工业增加值比重下降的态势。启动“出城入园”项目 93 个。实施重点节能和循环经济项目 50 个。国家级“两化”融合试验区通过工信部验收。完善商贸物流规划，健全市场体系，促进大众消费，商贸物流业增加值达到 351 亿元，占三产的比重达到 38.7%。文化产业增加值达到 41.06 亿元，增长 38.74%，占到生产总值的 2.31％。接待国内外游客 2606 万人次，增长28%，完成旅游总收入 206.5 亿元，增长 35％。积极鼓励全民创业，实施“三个 20 万”扶持小微企业发展政策，支持初创微型企业 1383 户，带动就业 8988 人。非公有制经济完成增加值 753 亿元，增长 23%，占生产总值的比重达到 43%。

（三）兰州新区基础设施建设和产业集聚实现较大突破。 坚持把全市工作重心向新区转移，市委市政府机关及部分市直部门和单位移至新区办公，集聚了人气商气，强化了“三区”融 合，全年共有 1800 批次、12500 名客商前来考察投资，新签招商引资项目 162 个，总投资 1826 亿元，到位资金 600 亿元。新区城市骨架初步形成，核心区 80 平方公里的水、电、路等基础设施基本到位，中川至马家坪铁路当年开工、当年建成，兰秦快 速路水阜至忠和段完成路基工程。产业集聚效应逐步显现，新 引进绿地集团、正威集团等 500 强企业，中国铁建、敬业农业等 17 个项目建成投产，四联光电、海默科技等 92 个项目加快建设，兰石集团、青岛啤酒等 30 个向新区拓展项目进展良好。学校、医院等公共服务设施加快配套完善。天然气管网和配套设施投入运行。污水处理厂和生活垃圾处理厂开工建设。新区内部和新区至市区 10 条公交线路开通运营。完成造林绿化 5 万亩，2 号生态湖等园林景观建成开放。工商、建设、交通、浦发等 10 家银行在新区设立分支机构，甘肃股权交易中心挂牌成立。一个西北地区重要的经济增长极、国家重要的产业基地、向西开放的重要战略平台、承接产业转移示范区正在快速崛起。

（四）城市功能形象显著提升。编制各类控详规划、专业规划和城市设计 30 多项，完成城市规划展览馆建设前期工作。实施未利用地试点开发，编制《未利用地专项规划》和《试验区总体规划》，完成土地开发 6.05 平方公里，积累了坪台地区开发建设的经验。全面加快轨道交通规划和实施工作， 完成试验段建设任务和 1 号线一期工程征地征收，全线开工准备就绪。南山路南出口以东建成通车，北环路基本完成路基工程，北绕城高速和南绕城高速开工建设，整治改造老旧道路 44 条。深安黄河大桥完成主体，雁白黄河大桥启动建设。小西湖立交改造工程建成通车，新建人行过街天桥 14 座。实施热电联产配套管网工程和东城区供热管网工程，敷设管线两条 30 公里。建成天然气北高压干线 20.6 公里，南次高压管线 11 公里，敷设中心城区管线 19 公里。新增、改造城区公共绿地 1367 亩。大力整治环境卫生、违法建设、占道经营等城市管理难题，拆除各类违法建设 50 多万平方米。完善“五城联创”工作方案，细化冲刺的目标、步骤和重点，“凡人善举、和你一起”活动作为创新案例入选中宣部培训教材。

（五）大气污染治理取得明显成效。在国家环保部的大力 支持和省委、省政府的精心指导下，我们坚持以人为本、科学 治污，标本兼治、狠抓落实，充分调动全市人民和社会各方面的 积极性，全力推进大气污染治理整体战攻坚战。投入各类资金26 亿元，完成城区 767 台 4347 蒸吨燃煤锅炉改造，实现了主城区燃煤锅炉及污染“双清零”。有效加强煤炭市场监管，整合 建成 2 个煤炭专营市场和 80 个二级营销网点。实施 33 个工业企业深度治理项目，淘汰落后产能企业 18 户。采取周边围挡、物料堆放覆盖、出入车辆冲洗、现场地面硬化、拆迁工地湿法作业“五个 100％”抑尘法，对城区所有施工工地实行了抑尘管控。全面禁止高排放车辆进入主城区，实施特殊条件下机动 车限行政策，全面停售低标号汽油，减少了汽车尾气排放。实 行道路机械化清扫、人工精细保洁和吸尘、清洗、洒水“五位一 体”作业模式。全面推行网格化管理，将近郊四区划为 1482 个网格，构建了覆盖主城区的城市管理网络。全年城区空气优良天数 299 天，优良率达到 82%，比上年增加 29 天。兰州退出了全国十大污染城市行列，这是自 2001 年有监测数据以来优良天数最多的一年，《人民日报》在头版头条先后以《兰州治污重 现蓝天》、《兰州掀掉雾霾“锅盖”》为题作了专题报道。大气环境质量的改善，明显促进了居民健康“，冬防”以来城乡居民呼吸系统疾病就诊病例和就医费用分别下降了三成左右。

（六） 扶贫攻坚带动了农村整体发展。积极落实省上

“1236”扶贫攻坚行动，制定了我市三年扶贫攻坚行动计划，确定了全面消除贫困村的建设目标，出台了贫困村脱贫考核标 准及验收办法。有效整合“双联”工作力量，强化“五级包抓”责 任制，最大限度集中联扶单位的优势和资源，投入资金 11 亿元，实施各类帮扶项目 537 个，农村发展在收入水平、富民产业、基础设施、生态环境、公共事业等方面取得了明显进步。新建农村公路 500 公里。解决 10 万人饮水安全问题。培训贫困农户 2.66 万人、“两后生”8200 人。协调发放惠农贷款3.4 亿元，惠及 5333 户，农村妇女小额担保财政贴息贷款1.17 亿元，惠及 1674 户。实现了城关、西固、红古三区整区脱贫和皋兰县一半贫困人口脱贫，完成 101 个贫困村整村脱贫，减少贫困人口 15 万人。全市农民人均纯收入达到 7114 元，增长 14.29%，净增 890 元，是多年来净增额较高的一年。

（七）民生保障和社会事业协调并进。完成为民兴办的实事 29 件，承办省上实事项目 23 件，累计投入资金 43.5 亿元。城镇居民人均可支配收入达到 20767 元，增长 12.6%。新增城镇就业 12.46 万人，城镇登记失业率控制在 1.71%。社会保险“五险合一”信息化应用平台上线运行。城乡低保标准分别提高 15%和 29%，惠及 19.22 万人。新建城市日间照料中心 62个、农村日间照料中心 73 个，虚拟养老院服务人数达到 22万人。建成残疾人综合服务中心，扶持残疾人稳定就业 1129人，培训 2439 人。分配入住保障性住房 10000 套，新开工建设14000 套，发放廉租住房租赁补贴 2769 万元。改造城市老旧住宅小区 165 个。改造农村危房 6090 户。完善住房公积金运营机制，当年归集资金 32.5 亿元，发放贷款 30 亿元。在全省率先实行义务教育阶段学生教辅资料免费发放，实施校车安全 全覆盖工程，开展了教师培训“千进八百互动计划”，新建城乡 公办幼儿园 73 所、寄宿制学校 127 所、标准化学校 88 所，引进北京实验二小在兰州合作办学。启动百名医师对口支援基 层医疗卫生工程，新建标准化村卫生室 15 个，新农合政府补助标准由 240 元提高到 290 元。继续实施五泉山、白塔山公园历史文化恢复工程。免费开放了水车博览园、文化体验馆和黄 河桥梁博物馆。新建 200 个城乡全民健身场地、105 个贫困村文化活动室。储备冬春蔬菜 1 万吨，积极开展平价肉菜进社区活动，为社区居民供应平价肉菜 8000 多吨。居民消费价格指数控制在 3.5%。完成食品药品监管体制改革。安全生产形势总体稳定，事故起数、死亡人数、致伤人数分别下降 0.35%、6.92%和 0.27%。完成 30 个标准化宗教活动场所创建任务，七里河区“两个共同”示范区创建工作深入推进。强化领导干部接待群 众来访机制，拓宽了信访渠道，化解了一批信访积案。依法打 击违法犯罪活动，着力化解矛盾纠纷，保持了社会和谐稳定。

（八）对外开放呈现新局面。坚持以开放促开发，推出“黄 河之都、金城兰州”城市形象新标志。继续强化城市营销，兰 州国际马拉松赛获得中国田协“金牌赛事”和国际田联“铜牌 赛事”称号。把招商引资作为扩大开放的重中之重，全面构建 大招商、招大商的工作格局，组建 160 个招商小组，确定 39个重点招商区域，全年赴外招商 6000 多人次，拜访企业

2000 多家。成功举办“兰洽会”和“民企陇上行”活动，组织参加了全国区域性重大节会活动，在兄弟城市开展招商推介活 动 42 场。强化产业招商、点对点招商和精准招商，新引进项目 925 个，签约金额 3528 亿元，到位资金 1350 亿元，增长70.77%。积极参与“丝绸之路经济带”建设，围绕“五通”，制定了打造经济带核心节点城市实施方案，着力推动 10 项重点工作，在北京举办了“丝绸之路经济带·兰州新区向西开放高 层论坛”，与哈萨克斯坦、罗马尼亚、塞尔维亚进行了多层次 交流合作，营造了向西开放的浓厚氛围。

（九）服务型政府建设不断加强。坚持依法行政，办理人大、政协议案、提案和建议 331 件，向市人大提交法规议案 6 件，制定政府规章 10 部。精简行政审批事项 171 项，保留 208 项， 兰州成为全国行政审批数量最少的省会城市之一。促进政务服务“两集中、两到位”，42 个部门的 304 项行政审批和服务事项进入市政务服务中心。整合成立了市公共资源交易中心。 建成全市电子监察系统，实现对行政审批、行政处罚、公共资源交易等事项的全方位监控。三维数字社会服务管理平台建成运行“，12345”民情通服务热线办理群众各类诉求 11.5 万件，成为全国信息化民生服务品牌。“民情流水线”工程被列为中央党的群众路线教育实践活动典型案例。严格落实中央八项规 定、国务院“约法三章”、省委“双十条”和市委“十四条”规定， 清理机关办公用房，全面禁止了楼堂馆所建设“，三公”经费下降 23.71%，各类会议精简 40%。深入开展“效能风暴”行动，加大督查和跟踪问责力度，各级干部积极投入治污治堵、招商引资、扶贫攻坚、征地拆迁、维稳信访和社会管理等工作一线，工 作作风和行政效能有了明显转变。军民融合、双拥共建活动深入开展，妇女儿童、老龄、残疾人、慈善等事业扎实推进，司法、 审计、统计、人防、气象、防震减灾等工作都取得了新进展。

各位代表！过去的一年，我们坚持解放思想、实事求是，坚持求真务实、少说多干，坚持勇于担当、积极作为，通过统 筹兼顾引领发展理念和方式的转变，通过深化改革开放激发 各类市场主体活力，通过创新体制机制提升社会民生保障水 平，取得了来之不易的成绩。这是省委、省政府亲切关怀和坚 强领导的结果，是市委统揽全局、科学决策和市人大、市政协 监督支持的结果，是全市人民众志成城、合力拼搏的结果。在 此，我代表市人民政府，向全市各族人民，各位人大代表和政 协委员，各民主党派、工商联、人民团体和无党派人士，向离 退休老同志和社会各界人士，向驻兰解放军指战员、武警官兵和公安干警，向所有关心支持兰州建设发展的朋友们表示 衷心的感谢和崇高的敬意！

我们也清醒地认识到，兰州经济社会发展和政府工作中还存在不少矛盾和问题。一是经济发展结构性矛盾突出。科技、教育、文化资源优势还没有充分发挥出来，产业内部结构不合理，一些不符合老城区功能定位的企业“出城入园”缓慢，园区、县区同质化发展现象突出。二是城市基础设施功能仍然薄弱。整体承载力与公共需求还不相适应，市区交通拥堵仍比较严重，背街小巷、城乡接合部、铁路沿线、河洪道环境治理滞后，违法建设、户外广告、“黑车”等痼疾顽症久治不愈。三是事关群众切身利益的问题亟待解决。教育、医疗资源配置不均衡，基层公共文化设施不完善，老龄事业发展滞后， 各类住房供给总量不足。四是县域经济实力较弱。农村基础设施欠账较多，农民持续增收的渠道不宽，新型城镇化和扶贫攻坚任务繁重。五是大气污染治理的长效机制尚未完全建立。部分单位和干部有厌战情绪，持续攻坚还要付出巨大努力。六是政府性债务管控力度需要进一步加大。部分融资平台、园区和县区融资成本较高，债务规模和偿债压力较大，潜在风险不容忽视。七是政府职能转变不到位。重审批、轻监管现象严重，一些部门和干部职工工作不扎实、效率不高，整治“四风”、加强廉政建设还要狠下功夫。对此，我们将坚持强烈的问题导向，不以事艰而不为，不以任重而畏惧，采取有效措 施，切实加以解决，让人民群众见到实实在在的成效。

2014 年主要任务

今年是深入贯彻党的十八届三中全会精神、全面深化改 革的开局之年，也是落实省委、省政府和市委决策部署、推动 我市转型跨越的关键一年。政府工作的总体要求是：高举中 国特色社会主义伟大旗帜，认真贯彻党的十八大以来各项部 署和习近平总书记系列重要讲话精神，积极落实“明地位、强 责任、作表率、出大力”的新要求，按照市委十二届七次全委 扩大会议暨全市经济工作和城镇化工作会议部署，坚持“稳中求进、改革创新”的总基调，深入实施“3341”项目工程，以加快发展、改善民生为努力方向，以招商引资、项目建设为抓 手，以作风建设、提高效能为保障，进一步解放思想、坚定信 心，咬定目标不放松，解决问题不懈怠，努力打造兰州经济社 会发展的升级版，当好全省科学发展、社会和谐的排头兵。

经济社会发展的主要预期目标是:生产总值增长 12%；规模以上工业增加值增长 11.5%；固定资产投资增长 27%；社会消费品零售总额增长 14%；公共财政预算收入增长 13%；城镇居民人均可支配收入增长 11%；农民人均纯收入增长 15%；城镇登记失业率控制在 4.6%以内；居民消费价格指数控制在3.5%以内；节能减排完成国家和省上下达的目标任务。兰州新区、高新区、经济区生产总值分别增长 30%、12%和 15.5%。

今年，重点抓好以下八个方面的工作：

（一）突出创新驱动，促进产业转型升级。始终把多元支 柱产业作为强市富民的根本支撑，充分发挥科技、教育、人 才、区位等综合优势，着力培育新的经济增长点，提升产业发 展实力和水平。大力发展农村富民产业。以更大的力度推动优势特色产业发展，新增高原夏菜 3.5 万亩、设施农业 1 万亩、玫瑰 2.5万亩。完成 54.5 万亩全膜双垄玉米和 54 万亩脱毒马铃薯种植，粮食产量稳定在 43 万吨。推广正大集团畜禽标准化生产经营模式，启动两个百万规模养殖项目，建设标准化规模养 殖场 100 个，力争全市畜禽养殖总量达到 930 万头只，畜牧业占农业比重达到 18%以上。扶持特色龙头企业 20 家，培育壮大重点农民专业合作社 30 个。鼓励农产品精深加工业发展，支持新建和续建项目 24 个。

积极培育发展战略性新兴产业。把握大数据时代特征， 用互联网思维提升产业层次，推动移动互联网、物联网、云计算等产业加快发展。深化“厅市会商”机制，以产业、政策、工作、 平台、人才和服务对接为重点，着力推进创新型试点城市、兰白科技创新试验区建设。完善兰州国家生物产业基地实施方案，建设“生物产业基地小微企业创新创业园”，放大兰州生物制品等 11 家骨干企业产学研功能。建成兰州重离子肿瘤治疗中心。推进信源软件园、联创智业园等特色项目建设。支持新材料新能源延伸开发，推动镍钴粉体材料、记忆合金等优势项目扩大生产规模。开展新能源汽车推广示范工作。规划申报国家自主创新试验区。举办首届“兰州科技成果交易周”。 力争战略性新兴产业增加值占生产总值的比重达到 7%。

促进传统产业改造升级。实施连海地区局域电网建设，推进煤电冶一体化发展，逐步打造有色冶金及深加工千亿元产业链。加快北车高端轨道交通装备制造等项目建设进度， 实施鸿丰电石等高载能企业技术改造。深入挖掘规模以上工业企业增长潜力，促进新入库 54 户企业增速达到 30%以上。建设远郊县区中小企业产业园区，促进中小企业集聚发展。 按照“消化一批、转移一批、整合一批、淘汰一批”的要求，化解部分行业过剩产能。
